# Syringomyelia: A Complication of an Underlying Pathology

**DOI:** 10.4021/jocmr2010.04.291w

**Published:** 2010-04-15

**Authors:** Mohammad Sami Walid, Mazen Sanoufa, Juan Salvatierra

**Affiliations:** a Medical Center of Central Georgia, Macon, Georgia, USA; b Mercer University, Macon, Georgia, USA

## Abstract

Patients with syringomyelia may have diverse etiology and experience a variety of symptoms. This report describes two cases of syringomyelia in patients with different profiles, presentations and pathomechanisms.

**Keywords:**

Syrigomyelia; Syrinx; Arachnoid cyst; Arnold-Chiari

## Introduction

Syringomyelia (syrinx) is a rare neurological disorder characterized by slowly developing fluid-filled areas that extend longitudinally down the spinal cord causing symptoms such as pain, weakness and stiffness in the back, shoulders, arms, and legs. Syringomyelia has a prevalence of 3.3 to 8.5/100,000 people with some ethnic variability [1-4]. In the United States, syringomyelia is more common in African-Americans than in Caucasians [5]. Patients may have diverse etiology and experience a variety of symptoms. This report describes two cases of syringomyelia in patients with different profiles, presentations and pathomechanisms.

## Case 1

**Figure 1.**
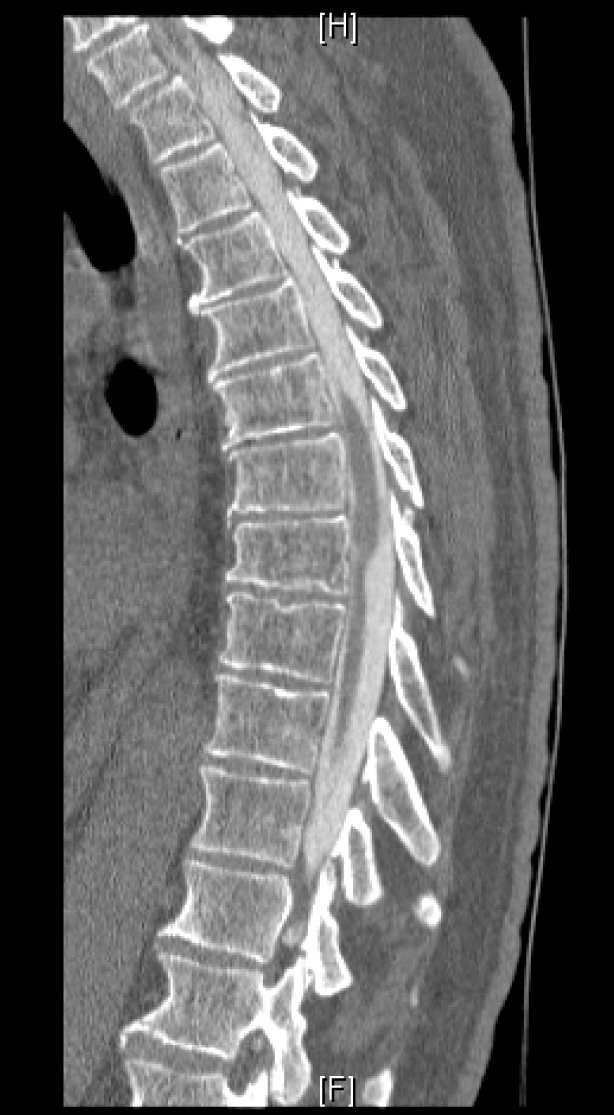
Computed tomography with contrast (a 67-year-old male patient).

A 67-year-old Caucasian male presents with a history of worsening leg numbness of several years duration. Imaging showed a T4-T8 syrinx. An arachnoidal cyst was suspected at T6 due to the flattening and anterior displacement of the cord immediately under the spinal cord enlargement (Fig. 1). The patient had no remarkable medical history and was on no medications. He denied bowel or bladder symptoms. He smoked cigarettes and drank alcohol daily. Neurological exam was unremarkable except for some decreased sensation to light touch below L1. The patient underwent T4 to T8 posterior decompression and exploration with removal of arachnoidal cyst and opening of syrinx.

## Case 2

**Figure 2.**
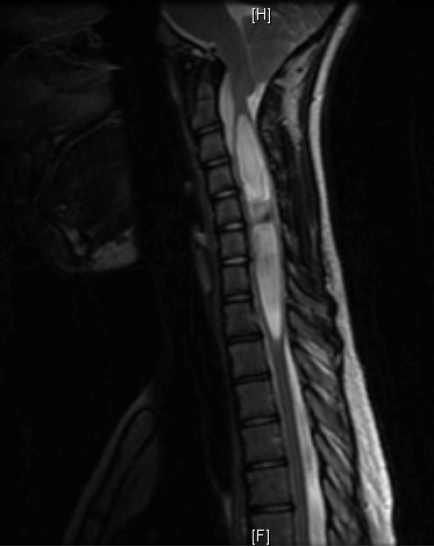
Magnetic resonance T2 image (a 12-year-old female patient).

A 12-year-old African-American girl presented with progressively worsening headaches. Neurological exam was unremarkable. Imaging showed a Chiari I deformity (low-lying cerebellar tonsils down to the level of C2) with a huge spinal cord syrinx from C2 to T2 (Fig. 2). Magnetic resonance angiography showed Limited flow through foramen magnum of the cerebrospinal fluid. The patient had posterior fossa craniotomy, C1-2 laminectomy and duraplasty for decompression of Chiari and midline cervical myelotomy for decompression and drainage of cervical syrinx.

## Discussion

Syringomyelia may be related to a congenital malformation or de novo abnormality in the central nervous system. Arachnoid cysts are intra-arachnoid collections of cerebrospinal fluid congenital in origin, however, onset of symptoms may be delayed until adolescence through expansion and pressure on the normal neural tissue leading to obstruction of the cerebrospinal fluid flow and neurological deficit [6]. These cysts may rarely be associated with syringomyelia or compressive myelopathy [7-9]. Spinal arachnoid cyst associated with syringomyelia can be treated by simple excision of the cyst without shunting the syrinx if the decompression effect resulting from removal of the cyst is sufficient [10].

Chiari malformation is another congenital abnormality whereby the cerebrellar tonsils herniate through the foramen magnum into the spinal canal causing disturbance in cerebrospinal fluid dynamics which may manifest by headaches, double vision, dizziness, and muscle weakness, particularly in the upper extermities. Most cases of non-traumatic syringomyelia occur in association with a Chiari malformation [11]. Very rarely both malformations (arachnoid cyst and Chiari malformation) can exist in a patient with syringomyelia [12, 13]. In most published cases the syringomyelia has been attributed to obstruction of cerebrospinal fluid flow at the foramen magnum by the arachnoid cyst itself. Very rarely, a posterior fossa arachnoid cyst may produce tonsillar descent and syringomyelia [12, 14].

Syringomyelia can be viewed as a complication of an underlying pathology which may be a simple arachnoid cyst or a complex Chiari malformation. It is important to keep these two entities in mind when dealing with patients expressing symptoms suggestive of progressive myelopathy in all age groups.
